# Association between the serum glucose-to-potassium ratio and clinical outcomes in ischemic stroke patients after endovascular thrombectomy

**DOI:** 10.3389/fneur.2024.1463365

**Published:** 2024-10-01

**Authors:** Qianqian Zhang, Zhihang Huang, Shuaiyu Chen, E. Yan, Xiaohao Zhang, Mouxiao Su, Junshan Zhou, Wei Wang

**Affiliations:** ^1^Department of Neurology, Nanjing First Hospital, Nanjing Medical University, Nanjing, China; ^2^Department of Neurology, Mianyang Central Hospital, School of Medicine, University of Electronic Science and Technology of China, Mianyang, China

**Keywords:** endovascular treatment, prognosis, GPR, large vessel occlusion, functional outcome

## Abstract

**Background and purpose:**

The baseline glucose-to-potassium ratio (GPR) is associated with poor outcomes in patients with acute brain injury and intracranial hemorrhage. However, the impact of serum GPR on clinical outcomes after endovascular thrombectomy (EVT) is unclear. This study aimed to evaluate the association between the GPR at admission and functional outcomes at 90 days after EVT.

**Methods:**

We retrospectively reviewed our database for patients with acute ischemic stroke involving an anterior circulation large-vessel occlusion who received EVT between October 2019 and December 2021. The baseline serum GPR was measured after admission. The primary outcome was a 90-day poor outcome, which was defined as a modified Rankin scale score of 3–6.

**Results:**

A total of 273 patients (mean age, 70.9 ± 11.9 years; 161 men) were finally included for analyses. During the 90-day follow-up, 151 patients (55.3%) experienced an unfavorable outcome. After adjusting for demographic characteristics and other potential confounders, the increased GPR was significantly associated with a higher risk of a 90-day poor outcome (odds ratio, 1.852; 95% confidence interval, 1.276–2.688, *p* = 0.001). Similar results were observed when the GPR was analyzed as a categorical variable. In addition, the restricted cubic spline observed a positive and linear association between the GPR and poor outcomes at 90 days (*p* = 0.329 for linearity; *p* = 0.001 for linearity).

**Conclusion:**

Our study found that ischemic stroke patients with the higher GPR at admission were more likely to have an unfavorable prognosis at 3 months, suggesting that GPR may be a potential prognostic biomarker for ischemic stroke after EVT.

## 1 Introduction

Stroke causes 5.5 million deaths annually and is the second leading cause of death worldwide (1), contributing to a growing global socioeconomic burden. Endovascular thrombectomy (EVT) has been confirmed to be beneficial for ischemic stroke patients with large-artery occlusion (2). Currently, the time window of EVT has been extended to 24h after stroke for patients with anterior circulation large vessel occlusion (3). However, the death and disability rates are still high. Therefore, predicting the outcome of patients following EVT early and accurately is important. Currently, serum biomarkers are used to predict outcomes of ischemic stroke, including those following EVT (4). These biomarkers help guide clinical decision-making for patients undergoing EVT.

Glucose is the main source of energy to maintain cellular metabolism (5). Several studies have shown that elevated glucose levels are associated with worse clinical outcomes in patients after EVT (6). Potassium plays an important role in physiological processes (7). In large vessel occlusion stroke, the ion composition, including the potassium, has been distinctly altered (8). Due to the complex interactions between glucose and potassium in physiological processes, the serum glucose-to-potassium ratio (GPR) has been utilized in a few studies. It has been shown to serve as an early prognostic factor for acute brain injury (9), intracranial hemorrhage (10), and neuropsychiatric syndrome after carbon monoxide poisoning (11). More recently, data from a Norwegian cohort demonstrated that increased GPR was associated with higher short-term mortality in ischemic stroke patients (12). However, to the best of our knowledge, it remains unknown whether the serum GPR is related to the prognosis of ischemic stroke in those receiving EVT. We therefore performed this study to evaluate the association between GPR and functional outcome at 90 days after EVT based on a retrospective cohort.

## 2 Materials and methods

### 2.1 Subjects and study design

This was a retrospective analysis conducted on a prospectively collected cohort of large vessel occlusive stroke patients who underwent EVT at the Nanjing First Hospital between October 2019 and December 2021. Patients were consecutively enrolled if they were (1) aged ≥18 years; (2) had acute anterior circulation large vessel occlusion in anterior circulation (internal carotid artery and middle cerebral artery: M1/M2 segments); and (3) had available data for calculating the GPR. To maintain the homogeneity of the enrolled patients, we excluded patients who were treated with intra-arterial thrombolysis alone and who used devices other than a stent-like retriever or aspiration system. The Institutional Review Board of the Nanjing First Hospital approved the study. All clinical investigations were conducted following the principles outlined in the Declaration of Helsinki. As the study was retrospective, patient consent could not be obtained but was waived. Patient data confidentiality was maintained at the Nanjing First Hospital.

### 2.2 Baseline variable assessment

Demographic characteristics, vascular risk factors, imaging data, and procedural characteristics were recorded during hospitalization. The baseline neurological deficit was measured using the National Institutes of Health Stroke Scale (NIHSS) by a certified vascular neurologist (13). Pre-treatment infarction volume was measured using the Alberta Stroke Program Early Computerized Tomography (ASPECT) score (14). Stroke etiology was classified based on the criteria of Trial of Org 10,172 in Acute Stroke Treatment (15). Collateral circulation was evaluated using the American Society of Interventional and Therapeutic Neuroradiology/Society of Interventional Radiology grading system, with grades 0–1 indicating poor collateral circulation and grades 2–4 indicating moderate to excellent (16). Successful recanalization was defined as modified thrombolysis in a cerebral infarction score of 2b or 3 (17). The sICH was diagnosed within 72 h of EVT using the Heidelberg Bleeding Classification (18, 19).

Before reperfusion treatment, blood samples were taken from all patients and immediately sent to the laboratory. GPR was obtained by dividing glucose level by potassium level (20).

### 2.3 Outcome measurements

Clinical information on patients’ outcomes after discharge was obtained prospectively during routine clinic visits or via telephone interviews with patients or their caregivers 3 months after the qualifying event. The primary outcome of this study was poor functional outcome, which was assessed using the modified Rankin Scale (mRS). An unfavorable outcome was defined as an mRS score of 3–6.

### 2.4 Statistical analysis

Quantitative variables were presented as mean ± SD or median (interquartile range), depending on the normality of the distribution, and categorical variables were presented as frequency (percentage). We utilized a *t*-test or Mann–Whitney *U*-test for continuous variables and a chi-square test or Fisher’s exact test for categorical variables. Logistic regression models were employed to investigate the odds ratio (OR) and 95% confidence interval (CI) of a 90-day poor outcome associated with each unit increase in GPR and across quartiles of GPR. Model 1 was adjusted for age and gender; model 2 was adjusted for model 1 and variables with a *p* < 0.1 in the univariate analysis including diabetes, baseline NIHSS score, pre-treatment ASPECTS, poor collateral status, successful reperfusion, sICH, and vessel occlusive site. Furthermore, restricted cubic splines (RCS) were used to explore the dose–response association between the GPR and clinical outcomes using three knots (at the 5th, 50th, and 95th percentiles) adjusted for covariates included in model 2 (21).

All statistical analyses were performed using SPSS, version 24.0 (IBM, Armonk, New York) and R (version 4.2.2). A two-sided *p* < 0.05 was considered statistically significant.

## 3 Results

### 3.1 Baseline characteristics

During the study period, 273 patients were admitted to the study in accordance with the inclusion criteria. The demographic characteristics, clinical features, and laboratory data of enrolled subjects are summarized in Table 1. The mean age was 70.9 ± 11.9 years, and 161 patients were men. The median baseline NIHSS score was 14.0, and the median pre-treatment ASPECT score was 9.0. Overall, 120 (44.7%) patients received intravenous thrombolysis before EVT. According to Heidelberg Bleeding Classification, 25 patients (9.2%) were classified as having sICH.

**Table 1:** Baseline data according to patients with and without poor outcomes at 90 days.

Variables	Total patients (*n* = 273)	Unfavorable outcome at 3 months		*P-*value
		Yes (*n* = 151)	No (*n* = 122)	
Demographic characteristics				
Age, years	70.9 ± 11.9	74.1 ± 9.9	66.9 ± 13.0	0.001
Men, *n* (%)	161 (59.0)	84 (55.6)	77 (63.1)	0.211
Risk factors, *n* (%)				
Hypertension	183 (67.0)	105 (69.5)	78 (63.9)	0.328
Diabetes mellitus	80 (29.3)	53 (35.1)	27 (22.1)	0.019
Hyperlipidemia	35 (12.8)	21 (13.9)	14 (11.5)	0.550
Current smoker	102 (37.4)	51 (33.8)	51 (41.8)	0.173
Coronary heart disease	39 (14.3)	26 (17.2)	13 (10.7)	0.123
Clinical data				
Systolic blood pressure, mmHg	136.3 ± 22.5	137.3 ± 23.3	135.1 ± 21.5	0.429
Diastolic blood pressure, mmHg	82.6 ± 14.1	82.9 ± 13.7	82.1 ± 14.6	0.624
Time from onset to recanalization, min	337.0 (230.0, 540.0)	351.5 (233.0, 564.0)	311.0 (222.0, 506.0)	0.250
Baseline NIHSS, score	14.0 (10.0, 18.0)	15.0 (12.0, 19.0)	12.0 (8.0, 15.0)	0.001
Baseline ASPECTS, score	9.0 (8.0, 9.0)	8.0 (8.0, 9.0)	9.0 (8.0, 10.0)	0.001
Stroke etiology, *n* (%)				0.105
Large-artery atherosclerosis	118 (43.2)	64 (42.4)	54 (44.3)	
Cardio-embolism	125 (45.8)	76 (50.3)	49 (40.2)	
Others/unknown	30 (11.0)	11 (7.3)	19 (15.6)	
Previous rt-PA treatment, *n* (%)	122 (44.7)	64 (42.4)	58 (47.5)	0.394
Poor collateral status, *n* (%)	134 (49.1)	84 (55.6)	50 (41.0)	0.016
Successful reperfusion, *n* (%)	242 (88.6)	126 (83.4)	116 (95.1)	0.003
sICH, *n* (%)	25 (9.2)	22 (14.6)	3 (2.5)	0.001
Location of the occlusive artery, *n* (%)				0.065
Middle cerebral artery	195 (71.4)	101 (66.9)	94 (77.0)	
Internal carotid artery	78 (28.6)	50 (33.1)	28 (23.0)	
Laboratory data				
GPR	2.26 ± 0.99	2.46 ± 1.13	2.00 ± 0.71	0.001
Hs-CRP, mg/L	8.5 (3.2, 22.9)	9.7 (3.2, 24.0)	7.4 (2.9, 20.2)	0.253

ASPECTS, the Alberta stroke program early computed tomography score; GPR, Glucose-to-potassium ratio; Hs-CRP, Hyper-sensitive C-reactive protein; NIHSS, National institute of health stroke scale; sICH, Symptomatic intracranial hemorrhage.

### 3.2 Association between GPR and clinical outcomes

Among the enrolled patients, the mean levels of GPR were 2.26. During the 90-day follow-up, 151 patients (55.3%) experienced an unfavorable outcome. On univariate analysis, compared to patients without poor outcomes, those with unfavorable outcomes were older (mean, 74.1 ± 9.9 versus 66.9 ± 13.0 years; *p* = 0.001). The prevalence of diabetes among patients with poor outcomes was higher than in patients without poor outcomes (35.1% versus 22.1%; *p* = 0.019). Patients with poor outcomes had lower baseline ASPECT scores (median, 8.0 versus 9.0; *p* = 0.014) and higher baseline NIHSS scores (median, 15.0 versus 12.0; *p* = 0.001). Baseline GPR was higher in patients with unfavorable outcomes than in patients without unfavorable outcomes (mean, 2.46 ± 1.13 versus 2.0 ± 0.71; *p* = 0.001). Poor collateral circulation (55.6% versus 41.0%; *p* = 0.016) and sICH (14.6% versus 2.5%; p = 0.001) were more common in patients with poor outcomes than in patients without poor outcomes. The successful reperfusion ratio in patients with poor outcomes was lower than in patients with favorable outcomes (83.4% versus 95.1%; *p* = 0.005).

**Figure 1:**
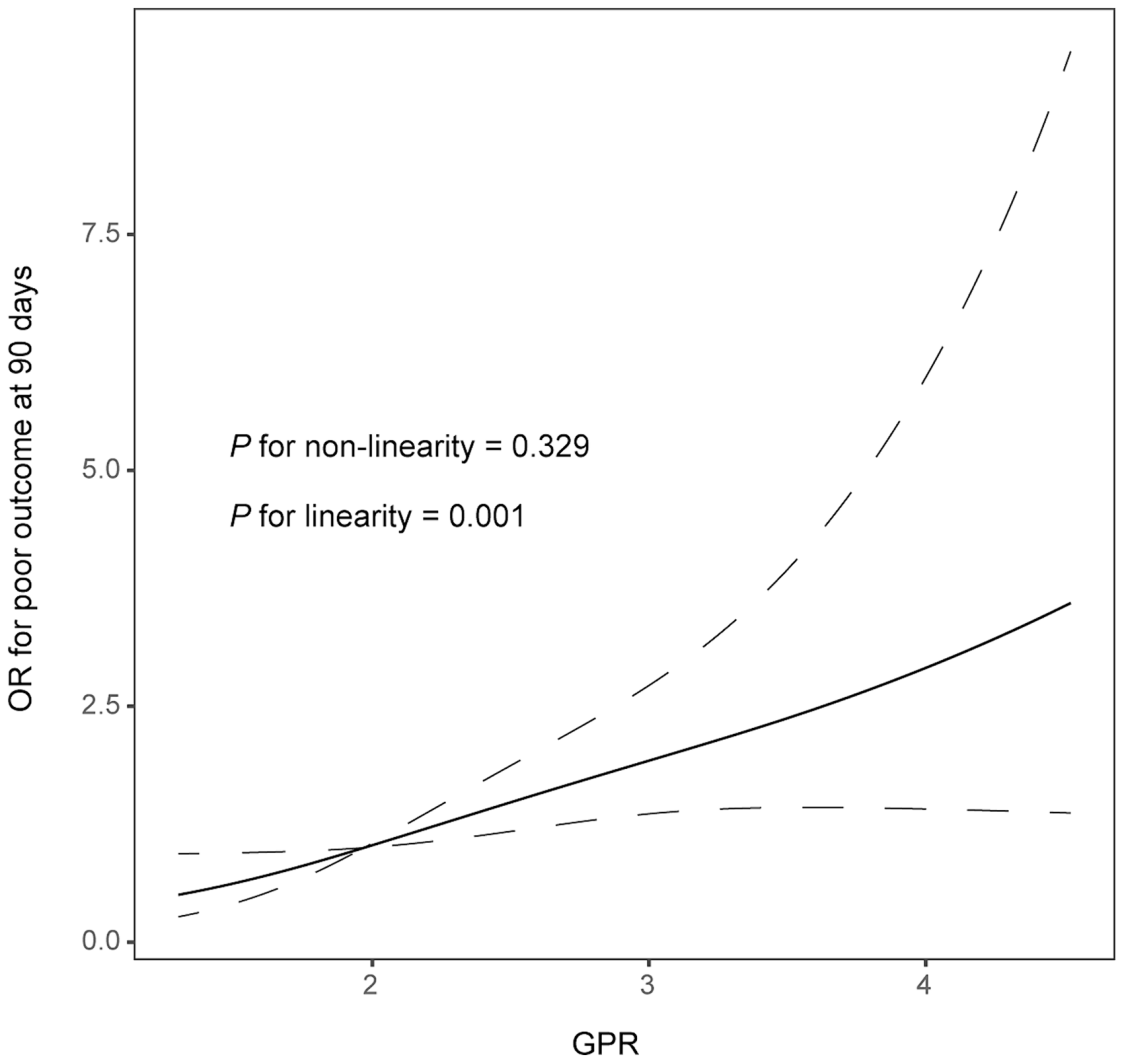
Correlation between GPR levels and risk of 90-day poor outcome. Odds ratio and 95% confidence intervals were derived from restricted cubic spline regression with three knots (at 5th, 55th, and 95th percentiles). The odds ratio was controlled for the same variables as model 2 in Table 2.

In multivariate logistic analysis after adjusting for potential confounders, increased GPR was significantly correlated with a higher risk of poor outcome at 90 days (odds ratio, 1.852; 95% confidence interval, 1.276–2.688, *p* = 0.001). Similar results were observed when the GPR was analyzed as a categorical variable (Table 2). In addition, the restricted cubic spline observed a positive and linear association between GPR and poor outcome at 90 days (*p* = 0.329 for linearity, *p* = 0.001 for linearity; Figure 1).

**Table 2:** Regression analysis for the relationship between GPR levels and 90-day poor outcome.

	Crude model		Model 1		Model 2	
	OR (95% CI)	*P-*value	OR (95% CI)	*P-*value	OR (95% CI)	*P-*value
GPR levels	1.810 (1.313–2.495)	0.001	1.926 (1.380–2.688)	0.001	1.852 (1.276–2.688)	0.001
GPR levels (quartiles)						
1st	Reference		Reference		Reference	
2nd	1.858 (0.936–3.686)	0.076	1.900 (0.922–3.919)	0.082	1.672 (0.752–3.718)	0.208
3rd	2.389 (1.212–4.708)	0.012	2.425 (1.186–4.960)	0.015	1.830 (0.831–4.030)	0.133
4th	3.564 (1.926–7.210)	0.001	4.104 (1.936–8.699)	0.001	3.201 (1.357–7.551)	0.008

CI, confidence interval; GPR, glucose-to-potassium ratio; OR, odds ratio. Model 1 adjusted for age and sex; Model 2 adjusted for demographic characteristics and variables with a *p* < 0.1 in the univariate analysis including diabetes, baseline NIHSS score, pre-treatment ASPECTS, poor collateral status, successful reperfusion, sICH, and vessel occlusive site.

## 4 Discussion

In this study, we found a prominent association between the GPR and clinical outcomes in ischemic stroke patients after EVT. Furthermore, the association still remained after adjusting for potential confounding factors. When the GPR was used as the categorical variable, the results showed an increasing trend of OR values from quartile 1 to quartile 4.

A previous study found that the GPR at admission is a promising predictor for 30-day mortality in ischemic stroke patients (12). In addition, the GPR is a potential predictor of prognosis for severe traumatic brain injury and intracranial hemorrhage (10, 22). Based on the published literature, the GPR has a close association between the Glasgow score and cerebral vasospasm in aneurysmal subarachnoid hemorrhage patients (23, 24). Furthermore, the GPR is evaluated as a predictive factor for the prognosis of acute intracerebral hemorrhage (10). The GPR is also related to the prognostication in severe traumatic brain injury requiring surgery, including acute subdural hematoma, acute epidural hematoma, traumatic brain contusion, and traumatic subarachnoid hemorrhage (25). In our study, the ischemic stroke patients after EVT with a higher GPR at admission were more likely to have an unfavorable prognosis at 3 months. However, the underlying pathological mechanism is not explained completely.

Post-stroke hyperglycemia is a type of stress hyperglycemia induced by high cortisol and catecholamine levels after ischemic injury (26). Regardless of diabetes status, hyperglycemia was independently associated with early stroke mortality (27). Previous studies revealed the association between the stress hyperglycemia ratio and 90-day clinical outcomes in patients with acute large vessel occlusion stroke receiving EVT (6, 28). However, another study found that stress hyperglycemia was associated with more severe strokes rather than directly predicting outcomes of acute ischemic stroke (29). In contrast, our study aligns with findings that patients undergoing EVT often experience more severe strokes and worse outcomes. Hyperglycemia involves an intricate and extensive physiological process that affects disease progression. Furthermore, serum potassium has a comprehensive function in maintaining the stability of the internal environment. Potassium plays a beneficial role in decreasing the risk of stroke (30, 31). The high cortisol induces low serum potassium by activating the renin–angiotensin–aldosterone system (RAAS). Johnson et al. (32) reported that serum potassium levels measured in early mid-life were linearly associated with the incidence of ischemic stroke, intracerebral hemorrhage, and all-cause mortality. A study in China has found that lower serum potassium levels are associated with the risk of recurrent acute ischemic stroke or transient ischemic attack (33). Hyperglycemia and hypokalemia reflect the activation of stress-related and hypothalamic–pituitary–adrenal (HPA) axis. According to previous studies, the GPR represents the activity of stress and the RAAS reaction. One of the physiological mechanisms of GPR to predict the 90-day outcome in ischemic stroke patients after EVT is stress, and the RAAS reaction produces excessive catecholamine secretion, elevates serum glucose level to promote secretion of insulin, and carries serum potassium into cells (34, 35). Therefore, a higher GPR indicates more serious damage to the body.

Although we identified the relationship between GPR and 90-day outcomes, there are several limitations in our study. First, as this was an observational study, we could not observe the direct association between GPR and 90-day outcomes in ischemic stroke patients after EVT. Second, we did not exclude unmeasured confounders affecting the GPR levels, including having infectious diseases prior to hospitalization. Thirdly, the levels of GPR were measured only once after admission. Finally, the study included the patients in a single center, which limits the applicability and generalizability of the findings.

In conclusion, the analysis demonstrated that GPR is a predictive index for 90-day outcomes in ischemic stroke patients who underwent EVT. These findings suggest that evaluating GPR may serve as an effective parameter for monitoring the outcome following EVT. Further studies with large sample sizes are needed to assess these associations comprehensively, which may help to identify patients at high risk of unfavorable outcomes after EVT and emphasize the importance of glucose and potassium after EVT.

## Data Availability

The raw data supporting the conclusions of this article will be made available by the authors, without undue reservation.
